# Exosomes: mediators of bone diseases, protection, and therapeutics potential

**DOI:** 10.18632/oncoscience.421

**Published:** 2018-06-23

**Authors:** Jyotirmaya Behera, Neetu Tyagi

**Affiliations:** ^1^ Department of Physiology, University of Louisville School of Medicine, Louisville, KY 40202, USA

**Keywords:** osteogenesis, angiogenesis, osteoclastgenesis, osteoporosis

## Abstract

Bone remodeling is a continuous lifelong process in the repair of micro-damage to bone architecture and replacement of aging tissue in bone. A failure to such process leads to pathological destructive bone diseases such as osteoporosis, rheumatoid arthritis, and osteoarthritis. However, this active process is regulated by; osteoclasts, which are involved in the bone resorption process; osteoblasts, with involvement in the bone formation process and bone-derived endothelial cells, which promote angiogenesis. In the bone micro-environment, these cellular interactions are mediated by a complex interplay between cell types via direct interaction of cell secreted growth factors, such as cytokines. Recently, the discovery of exosomes (∼ 40–100 nm in size), has attracted more attention in the field of the bone remodeling process. Exosomes and microvesicles are derived from different types of bone cells such as mesenchymal stem cells, osteoblasts, osteoclasts and their precursors. They are also recognized to play pivotal roles in bone remodeling processes including osteogenesis, osteoclastogenesis, and angiogenesis. In this review, we especially emphasize the origin and biogenesis of exosomes and bone cell derived exosomes in the regulatory process of bone remodeling. Moreover, this review article also focuses on exosomal secreted proteins and microRNAs and their involvement in the regulation of bone remodeling.

## INTRODUCTION

Age-related bone diseases such as osteoporosis, rheumatoid arthritis, and osteoarthritis are becoming the most universal and complex skeletal disorders worldwide [1-3]. They are characterized by disequilibrium between bone formation and bone loss upon aging and the inflammatory condition [3]. This imbalance of bone remodeling causes microarchitecture deterioration, bone fragility, and porosity as well as an increased risk of fracture [4, 5]. Thereby, the bone healing process is remarkably delayed, as evident from other studies in osteoporotic women and osteoporotic laboratory animals [6, 7]. Mechanistically, these skeletal defects are caused by a failure in the bone remodeling process through an imbalance in osteoclastic bone resorption and osteoblastic bone formation [1, 8]. Bone remodeling is a lifelong process, where mature bone tissue is replaced from the skeleton (by osteoclasts), called bone resorption and new bone tissue is then formed called ossification or new bone formation. These processes also control the reshaping or restoration of bone mass following injuries such as, fractures and skeletal inflammation but also micro-damage, which occurs during regular activity. This involves multiple and complex cellular and molecular events [9]. However, intercellular communication or paracrine signaling among these cell types are crucial for the establishment and maintenance of bone remodeling [10-12]. As osteoclasts play a pivotal role in pathological bone resorption; receptor activator of nuclear factor-κB ligand (RANKL) and macrophage colony‐stimulating factor (M‐CSF) are the key cytokines that induce osteoclastogenesis. Several master transcription factors, co-regulators, and morphogens play a pivotal role in regulating osteoblastogenesis. Several studies showed that factors such as Runx2, Osterix, Sox9, morphogens, TGFβ/BMP and FGFs are responsible for the terminal differentiation of osteoblast to bone mass phenotype [13, 14]. The tight co-regulation of bone resorption and bone formation is mediated by several secreted coupling factors linking these two cellular processes together. Semaphorin 4D (Sema4D) also known as CD100, expressed by osteoclasts, acts as a negative regulator of bone formation. Consistent with an inhibitory role for Sema4D on osteoblast-lineage cells, Sema4D inhibits bone mineralization *in vitro* [14, 15]. Other soluble factors such as sphingosine-1-phosphate, ephrins, and semaphorinsEphrinB2 are shown to play an essential role in cellular communication between osteoclast and osteoblast in the bone micro-environment.

Recent reports suggest that the cascade of the bone remodeling event is being regulated by critical factors that are packaged in lipid bilayered membrane vesicles called exosomes [16, 17]. Exosomes are small vesicles secreted by several cell types [18-21] and are suggested to play as essential mediators of intercellular communication [22-25]. At the present time, attention has been paid to exosomes in different pathophysiological settings. Growing evidence suggests that balanced bidirectional signaling between osteoclasts and osteoblasts mediated by exosome-transplantation overcomes bone loss due to pathological destructive bone diseases [17].

In bone milieu, exosomes regulate multiple cellular processes including bone cell differentiation and bone architecture maintenance via a paracrine manner [15]. Various studies have reported that bone cells [15, 26-28] release exosomes in the bone microenvironment, which facilitates a diverse cascade of intracellular or intercellular signaling mechanisms either by targeting same cells or neighbouring cells or reaching distant organs through circulation. Exosomes are small vesicles of endocrine origin, ranging between 40–100 nm in diameter and are released from multivesicular bodies with potential pro-osteogenesis capabilities [16, 17]. Thus, they act through a novel way to stimulate bone formation from different pathophysiological settings. Exosomes are produced from all mammalian cell types, carrying various functional bio-molecules including proteins, mRNAs, microRNAs and lipids and also play a crucial role in intercellular communications. Importantly, exosomes do not express cell surface major histocompatibility complex (MHC-I and MHC-II) proteins, and thereby overcome all the disadvantages over cell transplantation and therapy [25]. Exosomes can efficiently stimulate bone formation *in vivo* and *in vitro* [17]. In this review article, we highlight the role of bone-derived exosomes in the context of bone remodeling events by a coordinated balance of osteogenesis and osteoclastogenesis. Our review article also focuses on the diverse character of the exosome in bone marrow angiogenesis, as well as the intriguing therapeutic application of exosomes in different pathological destructive bone diseases.

## EXOSOME BIOGENESIS AND ITS COMPONENTS

**Figure 1:**
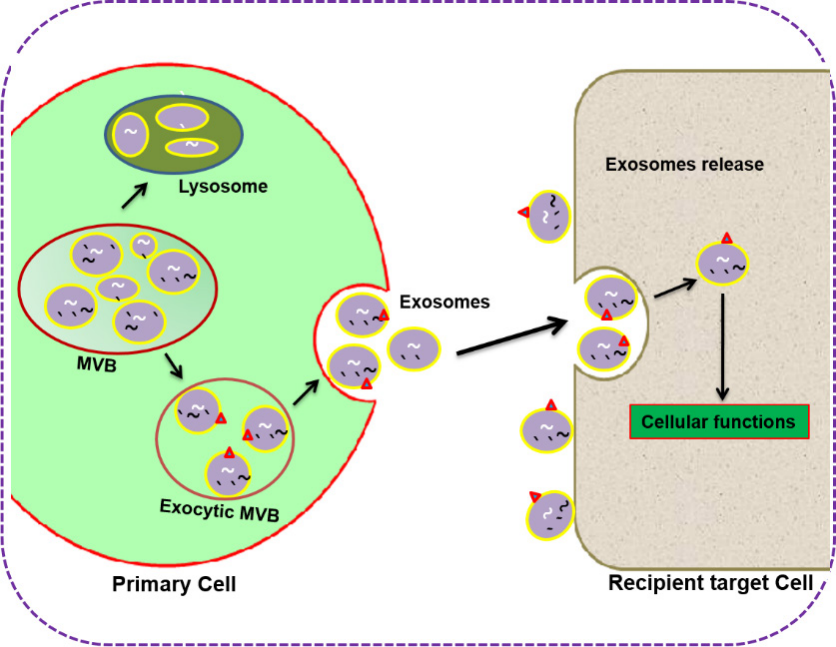
Biogenesis, secretion, and uptake of primary cell-derived exosomes in the target cells Exosomes are initiated by inward invaginations of clathrin-coated micro-domains on the plasma membrane and are converted into early endosomes (EE), carrying ubiquitinated cargos, facilitated by endosomal sorting complex required for transport (ESCRT). Then EEs, upon secondary invagination and maturation, convert into intraluminal vesicles (ILVs), which accumulate inside the endosomes called large multivesicular bodies (MVBs). The matured MVB can either be processed to lysosomes for degradation or fused with the plasma membrane (exocytic MVBs) for the release of ILVs into the extracellular space where it is called an exosome. Exosome secretion can be accelerated by various chemical, mechanical and environmental stimuli such as irradiation, low oxygen, and low PH. The exosomes secreted from primary cells will display various membrane components as their cells of origin. Following the release of exosomes, they may dock over the plasma membrane of recipient target cells. Furthermore, membrane-bound vesicles may either fuse with the plasma membrane directly or be endocytosed in the target cells. Upon endocytosis, exosomes may fuse with the delimiting membrane of an endocytic compartment and release its cargo contents, regulate the target cell gene expression, and finally cause cell commitment, differentiation, and activity.

It is well established that the content of exosomes varies from a diverse range of proteins, lipids, and nucleic acids. Understanding the intricacies of biogenesis and exosome trafficking and how crucial they are for intercellular communications and biological functions is the hot topic of current research. According to the study of Denzer et al. (2000), biogenesis of exosomes is initiated by inward invaginations of clathrin-coated microdomains on the cell membrane [45]. Following invagination, the invaginated vacuoles are converted into early endosomes (EE) that carry ubiquitinated cargos with the help of endosomal sorting complex required for transport (ESCRT). Then EEs, upon secondary invagination, form intraluminal vesicles (ILVs), which accumulate and mature inside the endosome that is now called large multivesicular bodies (MVBs) [30, 45, 46]. The mature MVBs now have two fates: either they can be processed to lysosomes for degradation or be fused with the plasma membrane (exocytic MVBs) for the release of ILVs into the extracellular space, [31] where they are then defined as exosomes (Figure 1).

Multiple studies have shown that the contents within exosomes are used as positive ‘markers’ for detection of exosomes from different origins. These proteins are membrane transport and fusion proteins (GTPases, flotillin, annexins), heat shock proteins (heat shock cognate (Hsc70) and (Hsp 90), tetraspanins (CD9, CD63, CD81 and CD82), proteins involved in MVB biogenesis (Alix and TSG101), lipid-related proteins and phospholipases [46, 47, 48]. However, the most widely used markers include TSG101, Rab5b, Alix and flotillin which are detected by Western protein expression and ELISA to confirm the presence of exosomes in extracellular body fluids or culture medium. To date, 4,400 different proteins have been identified that are associated with exosomes by mass spectrometry/proteomics analysis that serve as mediators in cell-cell intercellular communications [46, 48].

Exosomes are rich in a variety of lipids depending upon their cells of origin, and different types of exosomes have altered lipid composition. There is a variety of lipid compounds that are present in exosomes including phosphatidylcholine, phosphatidylethanolamine, phosphatidylserine (PS), lysophosphatidic acid, ceramide, cholesterol, and sphingomyelin [29]. Phosphatidylserine is involved in signal transduction and fusion to the plasma membrane by docking the outer proteins through its floppase, flippase and scramblase activities [49]. Lipids such as sphingomyelin and N-acetylneuraminic-galactosyl glucosylceramide (GM3) contribute different biophysical properties to exosomes and determine their rigidity and delivery efficiency [50]. Exosomes also contain nucleic acids in the form of miRNA, mRNA, and other non-coding RNAs apart from proteins and lipids [46, 51]. It is also reported that the RNA cargo of exosomes is different from that of the parent cell [46, 52]. However, cancer cells contain the same miRNA content as their parent cells which can be used as biomarkers [46, 50, 53]. The mRNAs enriched exosomes can be translated and cause mediation of biological functions in the recipient cells, while the miRNA and ncRNAs may activate transcriptional regulation of gene expression. The work of Koppers-Lalic et al. 2013, described that the functional RNAs present in exosomes are critical in the regulation of cell commitment, differentiation and activity [54]. In general, exosomes are generated from a wide range of cells and contain essential bio-molecules from their parent cells. Thus, exosomes serve as a shuttling transporter from their parent cells to target cells and mediate intercellular communication between cells. Observing the contents and considering major candidates can give credential to the bone remodeling potential of exosomes in bone disease. The main characteristics of extracellular vesicles are summarized in Table 1.

**Table 1:** Types of extracellular vesicles and their characteristics

Characteristics	Exosomes	Microvesicles	Apoptotic bodies
Size	40-100 nm	50-1000 nm	50-4000 nm
Morphology	Homogeneous cup-shaped	Heterogeneous irregular	Heterogeneous irregular
Origin	Endolysosomal pathway; multivesicular body	Cell surface; budding of cell membrane	Cell surface; blebbing of cell membrane
Morphology	Homogeneous cup-shaped	Heterogeneous irregular	Heterogeneous irregular
Buoyant density	1.12-1.22 g/cm3	None	1.17-1.29 g/cm3
Isolation method	Density gradient ultracentrifugation (100,000-200,000 g) and by immunoprecipitation (ExoQuick-INVITROGEN)	ultracentrifugation (10,000-80,000 g)	No proper standardized protocol
Molecular cargo	mRNA, miRNA, nc RNAs, mtDNAs	mRNA, miRNA, nc RNAs, mtDNAs	Nuclear fractions and cellular organelles
Possible markers	Tetraspanins (CD9, CD63, CD81, CD82), Alix, TSG101, HSP 70, flotilin-1	Integrin, CD40 metalloproteinase, Selectin, anexin V, flotilin-2	Phosphatidylserine and histones
Lipids	Ceramide, cholesterol, sphingomyelin and lysophosphatidic acid	Cholesterol	Phosphatidylserine
Biogenesis functions	Exocytosis of MVBs via ESCRT complex or sphingomyelinase and release through Rab-GTPase and SNAREs proteins	Budding and fission of plasma membrane through intracellular calcium	Membrane outward blebbing mechanism
References	[15, 17, 30-34, 134]	[23, 28, 35-40]	[41-44]

## ISOLATION AND PURIFICATION OF EXOSOMES

In the last decade, impressive research innovation has been made to isolate exosomes from biological tissues or fluids. To study the potential function of exosomes, the exosomes are specially isolated from cellular components. Various techniques are employed for isolation of exosomes such as differential ultracentrifugation, size-basedisolationtechniques(ultra-filtration), zonal rate centrifugation, immunoaffinity capture-based techniques, exosomal precipitation, and microfluidics-based isolation techniques, etc. [55, 56]. The isolated exosomes are composed of 40–150 nm vesicles characterized by electron microscopy, and both immunoblotting and flow cytometry (FACS) analysis based on exosome markers expression (such as Alix, TSG101, HSP70, etc.). The proteomic profiling approach is used to characterize the protein composition of exosomes present in it, and label-free spectral counting, to evaluate the effectiveness of each method in exosome isolation [57]. However, the majority of studies use a differential ultracentrifugation technique as the gold standard of exosomes isolation. By the use of specific markers like CD13, CD29, CD44, CD73, and CD105, exosomes were isolated from mesenchymal stem cells (MSC) [22, 58]. Until now, about 1069 proteins were identified in osteoblast (MC3T3 cell line) derived exosomes from the MSC origin through the expression analysis of exosomes marker flotillin 2 [59]. A total of 786 proteins are present in the ExoCarta database. A manually created database on exosomal proteins, RNA and lipids is available at ExoCarta (http://www.exocarta.org), which catalogs information from both published and unpublished exosomal studies. Currently, ExoCarta database (Version 3.1) contains information on 11,261 protein entries, 2375 mRNA entries and 764 miRNA entries that were obtained from 134 exosomal studies [60]. Exosomes from mature osteoclasts and its precursors are characterized by the presence of specific expression markers such as epithelial cell adhesion molecule 34 (EpCAM 34), tumor susceptibility gene (TSG) 101 and CD63 [61].

## MOLECULAR UNDERSTANDING OF BONE REMODELING AND ITS PATHOPHYSIOLOGICAL CONSEQUENCE

Bone remodeling is a complex, well-orchestrated process that occurs throughout life [62]. This complex coordinated event requires synchronized activities of multiple cell types to ensure bone remodeling (both bone formation and resorption) occurs sequentially to maintain bone mass [63]. A typical bone remodeling process happens within bone remodeling cavities and is performed by clusters of bone-resorbing osteoclasts, bone-forming osteoblasts and associated blood vessel-forming endothelial cells, which are arranged within temporary anatomical structures known as basic multicellular units (BMUs). These BMUs are covered by bone lining cells that form the bone remodeling compartment (BRC). Furthermore, BMC is interconnected with osteocyte lacunae, which is embedded in the bone matrix. This process begins with the initiation phase by bone-resorbing osteoclasts under the regulation of osteoclastogenic factors including RANKL and M-CSF, followed by bone-forming osteoblasts. However, this cycle is under the regulation of osteocytes and bone lining cell types [64]. Several coupling factors are also involved in osteoclast-mediated bone resorption, such as insulin-like growth factors (IGFs), transforming growth factor β (TGF-β), BMP, FGF and platelet-derived growth factor (PDGF) [4, 62]. To completely remove the damaged or infected bone, osteoclasts express a large family of glycoproteins sematophorin4D(Sema4D)thatbindtoPlexin-B1receptors in osteoblasts and inhibits IGF-1 pathway dependent osteoblast differentiation [65]. This phenomenon suggests that osteoclasts suppress bone formation by expressing Sema4D. Likewise, bidirectional signaling of ephrinB2/ephrinB4 promotes osteoblast differentiation and bone formation in the transition phase [66]. It has been reported that osteocytes act as orchestrators of the bone remodeling process despite osteoclasts and osteoblasts. Osteocytes release several factors such as sclerostin and DKK-1 that inhibit the osteoblast activity and stimulate local osteoclastogenesis [67, 68]. Sclerostin is a product of SOST and which antagonizes Lrp5, a vital receptor of the Wnt/β-catenin signaling pathway, in osteoblasts. For instance, bones subjected to pathological consequence, promote an increase in osteoclastogenesis via osteocyte expression, high RANKL/OPG levels and monocyte chemoattractant protein-1 (CCL2) expression [69]. Also, endothelial cells from capillary vessels are associated with the bone remodeling process by stimulating osteoblast activity within the BMUs. Therefore, vasculature located at the centre of BMUs may determine the process of bone resorption and bone formation. For the cortical remodeling, the capillary is provided through Haversian canals. However, the capillary supply for the trabecular remodeling comes from the marrow space [70]. The recent work suggests that endothelial specific Notch signaling promotes capillary outgrowth within the BMUs of the long bone. Genetic disruption of Notch signaling in endothelium in mice impaired vessel outgrowth and reduced osteogenesis and bone mass [70, 71].

The therapeutic potential of exosomes has been well documented in various organs and tissues such as the heart, lung, brain, and skin [72, 73, 74, 75]. These studies have provided an inspiring foundation for exosomal research. Although the outcome of exosomal research is encouraging, the exact underlying molecular mechanism of bone remodeling remain elusive. Moreover, recent reports suggest that exosome treatment enhances bone remodeling in both *in vivo* and *in vitro* models.

## ROLE OF EXOSOMES IN BONE PHYSIOLOGY

### Osteoclast exosomes: mediators of the bone remodeling process

The recent work of Huynh et al., (2016) reported that osteoclast precursor‐derived exosomes stimulate the differentiation ability of osteoclasts into mature phenotypes with significantly higher numbers than in the absence of exosomes. However, exosomes from osteoclast precursors promoted vitamin D dependent osteoclast formation in BM cultures, and exosomes from osteoclast-enriched cultures inhibited osteoclastogenesis [76]. The RANK level was enriched in exosomes from osteoclast cultures. Depletion of RANK during culture conditioning inhibits the exosome mediated osteoclast formation in vitamin D stimulated marrow cultures [76]. Therefore, this suggests, osteoclast-derived exosomes are paracrine regulators of osteoclastogenesis [76]. Recent research revealed that bone-derived exosomal micro RNAs (miRNAs) are involved in regulation of the bone remodeling process (Table 1). MicroRNAs (miRNAs) are small endogenous non-coding RNA molecules (containing ∼22 nucleotides), that are the key post-transcriptional repressors of gene expression. The exosomal derived novel miRNAs can modulate the differentiation and activities of osteoblasts and osteoclasts, by interacting with signaling molecules to control these processes. [77]. Mechanistically, the 5′ ends of mature miRNAs contain the seed region (nucleotide positions 2–7 or 2–8), which has the ability to silence the transcription of mRNA by specifically binding to its target sequence (complementary bases of the 3′-UTR). There are several miRNAs that have been characterized that act as negative regulators of bone formation. Osteoclast-derived exosomal microRNAs (miRNAs) represent a novel class of osteoclast-released coupling factors that cause inhibition of osteoblast differentiation. Increased osteoclastic miR-214-3p is associated with reduced bone formation in elderly women with fractures and ovariectomized mice [78]. Serum exosomal miR‐214 levels were also found to be significantly increased in osteoclast‐specific miR-214 transgenic mice [79]. Administration of chemically engineered oligonucleotides against miR-214-3p rescue the low bone formation phenotype in mice and in an *in vitro* osteoblast-osteoclast co-culture experiment [15, 78]. The work of Sun et al., identified that miR-214 is elevated in osteoclast exosomes and inhibits osteoblast activity via targeting EphrinA2/EphA2 interaction through co-culture experiments [15]. Similarly, miR-214 targets ATF4 in osteoblasts to inhibit bone formation [78]. The work of Zhao et al., (2015) suggested that miR-214 promotes osteoclastogenesis through PI3K/Akt pathways in BM macrophages [80]. These reports indicated that miR-214 containing exosomal osteoclasts mediate multifactorial effects that cause pathological destructive bone disease. Also, others have reported that exosomes secreted from monocytes (precursors to osteoclasts) stimulate osteogenic differentiation of MSCs [81, 82]. However, the molecular mechanism behind the osteoclast-mediated exosome activities that cause bone remodeling is a subject for future attention.

### Osteoblast exosomes: mediators of the bone remodeling process

The work of Deng et al., (2015) demonstrated that exosomes released from osteoblasts (UAMS-32P cell lines) contain RANKL protein and activate RANK signaling in osteoclast precursors through receptor ligand (RANKL-RANK) interaction, leading to osteoclast formation [83]. Such exosomes-mediated intercellular communication between osteoblasts and osteoclasts may represent a novel mechanism of bone remodeling. Exosomes from BM stromal cells can activate the bone remodeling process by enhancing osteoblast differentiation and mineralization. Growth factors, bone morphogenetic protein 9 (BMP9) and transforming growth factor‐β1(TGF‐β1) present in BM cell exosomes activate osteogenic differentiation [84]. The work of Solberg et al. (2015), described that osteoblast-derived lysosomal membrane protein 1 (LAMP1) positive exosomes carry RANK ligand, osteoprotegerin (OPG) and TRAP enzymes, which critically increase osteoclastogenesis [85]. Also, exosomes derived from mature osteoblasts also enhanced bone growth by up-regulating runt‐ related transcription factor 2 (RUNX2) and alkaline phosphatase, as well as enhanced matrix mineralization [86]. Cue et al. 2016, suggest that exosomal miRNAs are produced by mineralizing osteoblasts and promote osteogenic differentiation (ST2 cells). Osteo-miRNAs (miR-30d-5p, miR-133b-3p miR-140-3p, miR-335-3p, miR-378b and miR-677-3p) are highly expressed which mediate bone remodeling events, by regulating osteoblast differentiation and function through Wnt signaling, insulin signaling, TGF-b signaling and calcium signaling [86]. Functional evidence suggests that miR-30d-5p and miR-133b-3p inhibit the runt-related transcription factor 2 (RUNX2) gene expression, thereby inhibiting osteoblast differentiation [86]. Others have shown that miR-140-3p diminishes osteoblast activity by suppressing BMP-2 expression [88]. miR-677-3p was found to increase axis inhibition protein 1 (AXIN1) and enhance MSC osteogenic differentiation [86]. miR-378 activates the glucose-mediated osteogenic differentiation via the PI3K/Akt signaling pathway [89]. The prior reports also revealed that miR-335-5p increases osteoblast differentiation and mineralization via down-regulation of DKK-1 expression [90].

The work of Xu et al. (2014), reported for the first time the presence of miRNA in exosomes during BMSCs osteogenic differentiation [91]. They found that let-7a, miR-199b, miR-218, miR-148a, miR-135b, miR-203, miR-219, miR-299-5p and miR-302b were significantly upregulated in exosomes derived from BMSCs. However, miR-221, miR-155, miR-885-5p, miR-181a and miR-320c were significantly down-regulated in exosome samples [91]. Mechanistic studies revealed that microRNA, let-7, was reported to enhance bone formation by repressing adipogenesis in human MSCs through regulating HMGA2 gene expression [92]. The miR-218 and Wnt/β-catenin signal was reported to promote human adipose tissue-derived stem cells osteogenic differentiation via a signal-amplification circuit dependent manner [93]. miR-199b is also known to be involved in the control of osteoblast differentiation by Runx2 [92]. MicroRNA hsa-miR-135b could increase the somatic cells differentiation towards to osteoblast lineage [93]. Down-regulation of miRNA-221 in exosomes was reported to trigger osteogenic differentiation in human unrestricted somatic stem cells [91]. Wnt5a, a classical noncanonical Wnt, was reported as a critical component of BMP2 mediated osteogenesis. MiR-885-5p expression negatively regulates BM2-induced osteoblast activity by repressing Runx2 [91]. miR-181a represses TGF-Δ signaling molecules by inhibiting TΔR-I/Alk5 (TGF-Δ type I receptor) and accelerates osteoblast differentiation and mineralization [96]. Exosomal miRNAs are produced by osteoblasts and increase osteoclast activity via a paracrine mechanism. miR-148a was known to be upregulated in MSC exosomes, which further activates osteoclast differentiation and bone loss by targeting human V-maf musculoaponeurotic fibrosarcoma oncogene homolog B (MAFB) [97]. miR-503-3p from osteoblast-derived exosomes has been shown to inhibit RANK expression and RANKL-induced osteoclastogenesis [98]. In another study, human BMSC-derived extracellular vesicles are enriched with miR-196a which support bone formation in Sprague Dawley (SD) rats with calvarial defects [119]. Therefore, future research exploring the potential function of these exosomes-associated molecular factors and miRNAs, for example, paracrine/autocrine in communication between hBMSC/osteoblast or with other cell types in the field of bone remodeling, should bring new knowledge in this area. Collectively, the osteoblast-derived exosome and its molecular factors and miRNAs activate osteogenesis and bone remodeling by enhancing key osteoblast signaling molecules.

## EXOSOMES: POTENT ANGIOGENIC FACTORS THAT PROMOTE ANGIOGENESIS

**Figure 2:**
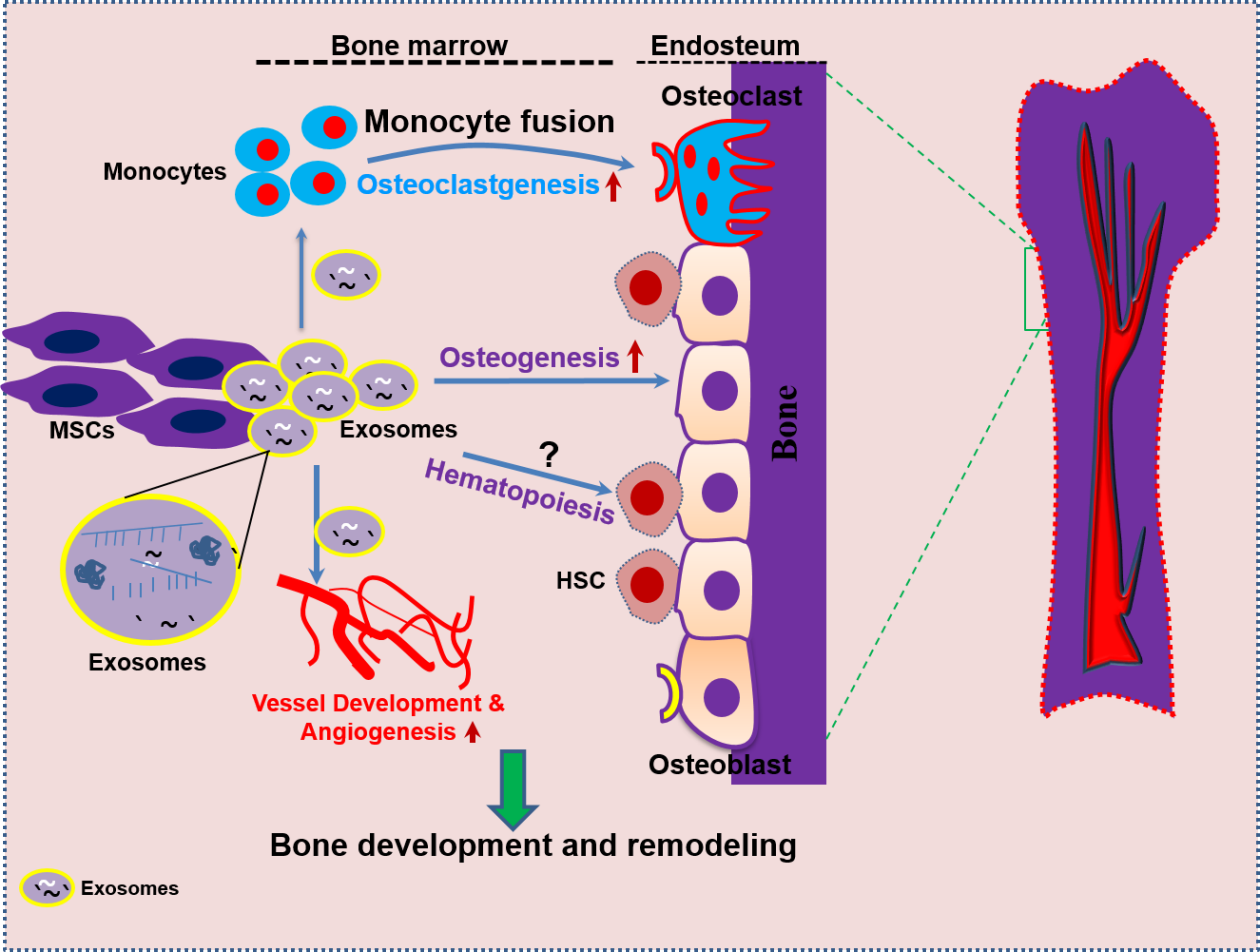
Bone marrow-MSC derived exosomes enhance bone regeneration by orchestrating a coordinated regulation of osteogenesis, angiogenesis, and osteoclastogenesis In the bone microenvironment, bone marrow-MSCs actively secrete exosomes, which are taken up by the surrounding cells including osteoblasts, osteoclasts, and endothelial cells. These activities result in a complex interplay of bone homeostasis by accelerating osteogenesis, osteoclastogenesis, and angiogenesis of which may promote vascularized bone development and regeneration.

For a skeleton to sustain its bone mass growth and development, it has to obtain greater amounts of oxygen and nutrients through the formation of new blood vessels or angiogenesis. Angiogenesis refers to the formation of new capillaries or vessels from existing blood vessels mediated by an orchestra of a multistep process of cellular events [98, 100, 101]. Several studies reported that blood vessel development occurs through the active involvement of soluble growth factors [Fibroblast Growth Factor] (FGF) and Vascular Endothelial Growth Factor (VEGF) associated with endothelial cell growth and differentiation [99, 102], inhibiting factors (angiogenin) for proliferation and stimulating differentiation of endothelial cells [99, 103] or extracellular cytokines (angiostatin and endostatin) [104]. Mass spectroscopy analysis of exosomes profiling revealed that MSCs derived exosomes contain soluble growth factors such as VEGF, TGFB1, interleukin-8 (IL-8) and are rich in transcription factor (HGF), which accelerate the pro-angiogenic activity by stimulating both proliferation and migration of endothelial cells [105, 106]. Similarly, human T-cell factor 4 (TCF4) is a key effector of Wnt signaling, a canonical pathway that exerts a central role in vessel development [107]. Therefore, intercellular transmission of exosomes containing HGF, HES1 and TCF4 factors may have both proangiogenic and pro-survival effects in organizing vascular phenotypes. Also, the work of Chen et al. (2010), reported that exosomes derived from MSCs contain miRNAs, including miR210, miR126, miR132, and miR21, which are shown to be involved in angiogenesis [106] and miR-6087 which induces endothelial differentiation [108, 109]. A recent research report has shown that exosomes from MSCs successfully accelerated angiogenesis in different *in vivo* animal models. The work of Bian et al. (2014), reported that bone marrow MSCs derived exosomes promotes angiogenesis in the ischemic heart by reducing myocardial ischemic/reperfusion injury in rat models [22]. In another study, it was also revealed that umbilical cord derived-MSC exosomes attenuated hind-limb ischemia by promoting blood flow [28]. In another study, exosomes derived from bone marrow tumor cells (myeloid leukemia cell line K562) were enriched with a large amount of miR-92a that enhanced angiogenesis under normoxic and hypoxic conditions [110]. With chronic hypoxia, exosomes secreted by multiple myeloma cells also improve angiogenesis by targeting factor-inhibiting hypoxia-inducible factor-1 via miR-135b. Sahoo et al. (2011), reported that exosomes from mobilized human CD34+ cells are enriched with miR-126 and miR-130a which enhances endothelial tube formation *in vitro*. Moreover, *in vivo* studies showed that the CD34+-exosomes stimulated angiogenesis in Matrigel plug assays [111]. Exosomes secreted by HMSCs, attenuate hindlimb ischemia by promoting endothelial activity and angiogenesis in mice [112]. The most recent work of Qi et al. (2016), reported that exosomes from MSCs derived from human induced pluripotent stem cell (hiPS) could promote bone regeneration in critical size bone defects in an ovariectomized rat model by enhancing both bone formation and angiogenesis [113]. This study reveals that exosomes from hiPSC-MSC accelerate significantly more neovascularisation by increasing vessel area and vessel number by enhanced osteoblast alkaline phosphatase (ALP) activity and bone formation markers (RUNX2 and COL1). Therefore, angiogenesis is only one of many intriguing effects of cell derived exosomes which have been associated with vascular capillary network formation for tissue or organ regeneration. This suggests that exosomes may be novel mediators to be employed in the treatment of various diseases. The detailed mechanism by which exosomes modulate angiogenesis in the bone microenvironment remains incompletely understood. Therefore, more research is warranted to explore the exosome mediated blood vessel development or angiogenesis that leads to the development of novel treatment for pathological destructive bone disease and remodeling. The overall feature of bone cell-derived exosomal secreted factors and miRNAs and its involvement in the bone remodeling process is depicted in Table 2. Moreover, the exosomal role in the regulation of bone development and vascularization has been illustrated in (Figure 2).

**Table 2:** Bone cell-derived exosomal secreted factors, miRNAs, and their involvement in the bone remodeling process

Source	Exosomes containing Secretary Factors/miRNAs	Biological functions	References
Osteoclast	RANK	Stimulate osteoclasts and osteoblastic differentiation in bone	[74]
Osteoclast	EphA2, B2	Osteoblast differentiation	[76, 66]
Osteoclast	IGF,	Activate osteoblast migration	[77]
Pre-osteoclast	PDGF	Promotes angiogenesis by specific endothelial (CD31+EMCN+) cell types	[78]
Osteoclast	Cardiotropins-1	Accelerates osteoblast differentiation and mineralization	[79]
Osteoblast	RANKL	osteoclast formation and activity	[83]
Osteoblast	OPG	Inhibit the osteoclast differentiation through OPG-RANKL interaction	[17, 83]
Osteoblast	TRAP	Increasetheosteoclastgenesis	[17, 85]
Osteoblast	PP1C and PABP	Regulate EIF2 signaling pathway in osteogenesis	[86, 87]
MSCs	Undefined factors?	Promotes osteogenesis and angiogenesis	[115]
Osteoclast	MiRNA-214	inhibits the osteoblast activity via targeting EphrinA2/EphA2 interaction and also targets ATF4 to inhibit bone formation	[15, 78]
HBMSCs	miR‐135b	Inhibits osteoblast differentiation by targeting IBSP and Osterix	[94, 95]
HBMSCs	miR‐885‐5p	Inhibits osteogenic differentiation by targeting RUNX2	[91]
HBMSCs	miR‐181a	Increases osteoblast activity and mineralization through TGF‐BI regulation	[96]
HBMSCs	miR‐218	Accelerates osteoblast differentiation and mineralization through Wnt signaling	[131]
HBMSCs	miR‐196a	Increases osteoblast differentiation and mineralization by targeting HOXC8	[119, 133]
HBMSCs	miR‐148a	Increases the osteoclast differentiation through by targeting V‐maf musculoaponeurotic fibrosarcoma oncogene homolog B	[92, 97]
HBMSCs	let‐7	Increase osteogenesis and bone formation by HMGA2	[92]
Osteoblast	miR-503-3p	Attenuating osteoclastgenesis by targeting RANK receptor	[98]
Osteoblast	miR‐133b‐3p	Attenuating osteoblastgenesis by targeting RUNX2	[132]
Osteoblast	miR‐30d‐3p	Attenuating osteoblastgenesis by targeting RUNX2	[87]
Osteoblast	miR‐677‐3p	Promotes MSC osteogenic differentiation via targeting AXIN1	[86]

## EXOSOMES: MEDIATORS OF SKELETAL MUSCLE REGENERATION

Mesenchymal stem cell (MSC) transplantation is widely used for the treatment of various disease models due to its paracrine effect in tissue regeneration. Recently, exosomes have attracted special attention as new players in cell-to-cell communication for tissue regeneration. The work of Nakamura et al. (2015) has reported that MSCs-derived exosomes can promote skeletal muscle regeneration by enhancing myogenesis and angiogenesis in a muscle injury model [116]. A cytokine antibody array revealed that significant amounts of angiogenic factors such as VEGF and IL-6 are present in the MSCs derived exosomes that contribute to the regeneration of skeletal muscle [114]. On the other hand, among others, miR-494 is contained abundantly in MSC-exosomes, and the results have shown that miR-494 participates in C2C12 muscle myogenesis and endothelial migration activity. miR-24 is involved in myogenesis via modulation of transforming growth factorβ, myogenin and MEF2 [115]. Furthermore, miR-181 is upregulated during muscle differentiation, which targets homeobox AII, a repressor of the differentiation process [116]. Thereby, it enhances muscle growth. Exosomes including both miRNAs and their secretion of cytokines or growth factors from MSCs may explain the mechanism of skeletal muscle regeneration during MSC transplantation and may be a new therapeutic tool.

## ROLE OF EXOSOMES IN BONE FRACTURE

It has been reported that exosomes play as important mediators, which transfer genetic material (miRNA, mRNAs), proteins and lipids to target cells [41, 46]. Some studies have defined stem cells, or their precursor's cells derived exosomes as being involved in a bone remodeling and repair mechanism [115, 118]. The work of J. Xu et al. (2014), reported for the first time that BMSC derived exosomes are enriched in let-7a, miR-199b, miR-218, miR-148a, miR-135b, miR-203, miR-219, miR-299-5p, and miR-302b, that are known to induce osteogenesis by regulating RNA degradation, the mRNA surveillance pathway, Wnt signalling pathway and RNA transport mechanism [91]. Similarly, Furuta et al. (2016), also supported the role of exosomal miRNA (miR-21, miR-4532, miR-125b-5p and miR-338-3p) and bone repair related cytokines (MCP-1, -3, SDF-1 and angiogenic factors) in osteogenic bone formation in the CD9−/− mouse model [117]. These enhanced fracture-healing phenotypes with bone remodeling events are due to the well-orchestrated process of osteogenesis and vascular angiogenesis. J. Zhang et al. (2016), proposed that exosome/tricalcium phosphate (β-TCP) scaffold-mediated pro-osteogenesis effects on hBMSCs towards osteogenic lineage via pi3k-Akt signaling pathways [118]. They showed that naturally secreted nanocarriers-exosomes could act as biomaterials and potentially enhance bone repair and remodeling. In another study, BMSC-derived exosomes were found to be enriched in osteogenic-related miRNAs, miR-196a, miR-27a and miR-206, which were highly upregulated. They further confirmed these findings with *in vitro* functional studies [119, 120].

## ROLE OF EXOSOMES AS CLINICAL THERAPEUTICS IN BONE DISEASE

Due to several cellular and molecular traits like self-renewal, vast differentiation and a variety of ECM protein secretion, MSCs have been proposed to be an important candidate for tissue repair, especially for mass bone restoration. However, several therapeutic findings of MSCs or its precursors-derived exosomes have been reported in the field of orthopaedics [121, 122, 123]. However, debilitating bone disease such as osteoporosis, rheumatoid arthritis (RA) and osteoarthritis brought significant attention in clinics to recover patients from inflammation, T-cell activation, and imbalanced bone remodeling, which lead to pain and deterioration of bone mass and joints. However, there is no direct study on the role of stem cell-derived exosomes that mitigate the imbalance in the remodeling process in the aforementioned destructive bone diseases. The work of Zhang et al. (2015), reported that exosomes released from human iPS-derived MSCs improve cutaneous wound healing in a murine model by enhancing collagen synthesis and angiogenesis [124]. In another study, ESC-derived exosomes improved cardiac function by strengthening myocardial neovascularization via a miRNA-290–295 dependent manner following myocardial infarction [125]. The work of Nakamura et al. (2015), also reported the emerging role of MSC-derived exosomes in being able to accelerate skeletal muscle regeneration via miRNA-494 in a skeletal muscle injury model [116]. Bone vascularization is essential for many physiological processes, such as bone development and growth, and bone remodeling. The work of Qi et al. (2016), also reported that MSCs derived exosomes from hiPS can promote bone formation and vascularization in critical size bone defects in an ovariectomized rat model [115]. Interestingly, in a murine model of delayed-type hypersensitivity and collagen-induced arthritis, EVs containing adenovirus expressing viral IL-10 or bone marrow-derived DCs treated with recombinant murine IL-10 suppressed the inflammatory and autoimmune responses when injected particularly [126]. This may represent a novel, cell-free therapy for the treatment and has major applications in the field of osteobiology and orthopaedics. However, if such type of MSCs or BM-derived exosomes could be isolated on a large scale in clinical practice that could be a major therapeutic intervention in debilitating bone diseases, including osteoporosis, rheumatoid arthritis, and osteoarthritis.

## THERAPEUTIC ADVANTAGE OF EXOSOMES TREATMENT AND FUTURE DIRECTIONS

Much of the excitement surrounding extracellular vesicles/exosomes research is due to its high clinical relevance. This is due, in particular, because bone exosomes can be easily isolated through minimally invasive procedures, such as from the bone marrow or MSCs of healthy donor patients, as they have great potential in destructive bone disease diagnosis. Also, exosome treatments have several advantages due to fewer safety concerns over cell-based treatments and reduce toxicity and immunogenicity problems [115, 127]. Also, exosomes do not express cell surface MHCI or MHCII proteins and thereby prevent immunogenicity better than cell-based transplantation therapy [128] which can effectively stimulate bone remodeling *in vivo* and *in vitro* [124]. MSC-derived exosomes also maintain their privileged immune properties of their origins, and this may significantly help researchers to develop novel immunotherapies [129]. Additionally, in comparison with living cells, nonviable exosomes are more stable, have no risk of aneuploidy and a low possibility of immune rejection following *in vivo* administration [75]. Multiple prior studies have reported the use of MSCs and miRNAs in bone repair and remodeling [121-123, 130]. The above findings suggest that stem cell-derived exosomes will one day be able to provide the best possible clinical medicine in the field of bone repair and remodeling therapy. Since MSCs derived exosomes accomplish such recovery tasks, further research will be needed to identify a novel exosome; thereby efficiently recovering the bone remodeling phenomena in the *in vivo* condition. Therefore, they can provide an alternative therapy for bone and other diseases.

## CONCLUSIONS

In conclusion, our review article collectively focused on the recent approaches towards the therapeutic application of exosomes in the bone remodeling process, which regulates osteoclastogenesis, osteoblastogenesis, and angiogenesis. However, the molecular mechanism behind the exosomes mediated signaling cascade in bone remodeling and development remains elusive. Biomedical application of exosomal based medication will bring a new challenge in clinical practice. To improve such clinical conditions, at the very least, novel reliable methods must be developed for easy purification of exosomes in large-scale production, and for co-expression of different molecules (proteins, mRNA, and miRNA) that affect physiological function and its administration route needs to achieve targeted delivery and recovery of pathological outcomes to be determined. Considering the enormous importance of exosome-based clinical therapy, it could be a new and safe approach for debilitating bone diseases more so than other gene and cell-based therapies.

## Abbreviations

ESCRT: Endosomal sorting complexes required for transport
SNARE: Soluble NSF Attachment Protein Receptor
EMCN: Endomucin
IBSP: Integrin binding sialoprotein
